# Allergen-specific IgA and IgG antibodies as inhibitors of mast cell function in food allergy

**DOI:** 10.3389/falgy.2024.1389669

**Published:** 2024-06-11

**Authors:** Kameryn N. Furiness, Yasmeen S. El Ansari, Hans C. Oettgen, Cynthia Kanagaratham

**Affiliations:** ^1^ Division of Immunology, Department of Pediatrics, Boston Children’s Hospital, Boston, MA, United States; ^2^ Institute of Laboratory Medicine, Philipps University Marburg, Marburg, Germany; ^3^ Department of Pediatrics, Harvard Medical School, Boston, MA, United States

**Keywords:** mast cells, IgA, IgE, IgG, food allergy, Fc receptor, oral immunotherapy

## Abstract

Food allergy, a group of adverse immune responses to normally innocuous food protein antigens, is an increasingly prevalent public health issue. The most common form is IgE-mediated food allergy in which food antigen-induced crosslinking of the high-affinity IgE-receptor, FcεRI, on the surface of mast cells triggers the release of inflammatory mediators that contribute to a wide range of clinical manifestations, including systemic anaphylaxis. Mast cells also play a critical function in adaptive immunity to foods, acting as adjuvants for food-antigen driven Th2 cell responses. While the diagnosis and treatment of food allergy has improved in recent years, no curative treatments are currently available. However, there is emerging evidence to suggest that both allergen-specific IgA and IgG antibodies can counter the activating effects of IgE antibodies on mast cells. Most notably, both antigen-specific IgA and IgG antibodies are induced in the course of oral immunotherapy. In this review, we highlight the role of mast cells in food allergy, both as inducers of immediate hypersensitivity reactions and as adjuvants for type 2 adaptive immune responses. Furthermore, we summarize current understanding of the immunomodulatory effects of antigen-specific IgA and IgG antibodies on IgE-induced mast cell activation and effector function. A more comprehensive understanding of the regulatory role of IgA and IgG in food allergy may provide insights into physiologic regulation of immune responses to ingested antigens and could seed novel strategies to treat allergic disease.

## Introduction

Food allergy is a severe and potentially life-threatening health condition affecting the lives of approximately 8% of children and 2%–3% of adults in the United States (1). As an immunological disorder, food allergy results from the breakdown of oral tolerance, a state of unresponsiveness to ingested soluble antigens (2). The most common form of food allergy is IgE-mediated and is characterized by the production of food-specific IgE antibodies, which activate mast cells (MCs) and basophils upon ingestion of food antigens.

As the key effector cells of anaphylactic reactions, MCs play a critical role in the pathogenesis of food allergy. They reside in the skin and mucosal tissues where they are among the first cells of the immune system to interact with foreign antigens. MCs express the high affinity IgE-receptor FcεRI on their surface (3). Antigen-induced crosslinking of IgE antibodies bound to this receptor results in the release of potent, preformed inflammatory mediators contained in granules, the rapid synthesis of eicosanoids, and the *de novo* production of type 2 inflammatory cytokines (4–7). Following their release, these mediators act on various target tissues to trigger both local and systemic physiological responses, including nausea, abdominal pain, vomiting, hives, angioedema, difficulty breathing, and decreased cardiac output along with intravascular volume loss manifesting as shock (2). Systemic anaphylaxis, the most severe manifestation of food allergy, is characterized by multi-organ system involvement, including the skin, respiratory system, cardiovascular system, and central nervous system (8).

While the primary focus of this review is the role of MCs in the pathogenesis of food allergies, it is important to note that basophils can also contribute to the allergic phenotype. Like MCs, IgE-mediated aggregation of FcεRI on basophils results in the release of their granule contents along with rapid production of prostaglandin and leukotriene mediators (9). Both MCs and basophils arise from CD34+ hematopoietic progenitors; however, MCs are long-lived, tissue-resident sentinel cells, while basophils are short-lived cells that mature in the bone marrow and circulate in the blood (9). Because basophils are not abundant in the intestinal mucosa at baseline, they are not typically considered as instigators of immune sensitization or effectors of acute reactions in food allergy (10).

Presently, there is no cure for food allergy. Some of the cutaneous manifestations of IgE-mediated reactions to foods, including pruritus, urticaria, and angioedema, can be alleviated using antihistamines; however, antihistamines are ineffective in treating anaphylaxis and do not attenuate the T-helper type 2 (Th2) immune response to food proteins (9). For any but mild cutaneous reactions, intramuscular epinephrine injection is a useful therapy. The only existing disease-modifying treatment is immunotherapy (IT), which can be sublingual (SLIT), epicutaneous (EPIT), or oral (OIT). Currently, peanut powder (*Arachis hypogaea* allergen powder-dnfp) OIT is the only FDA-approved immunotherapy treatment, although studies have confirmed the efficacy of OIT to desensitize children with allergy to a number of other common allergenic foods in addition to peanut (11, 12). OIT refers to the oral administration of increasing amounts of allergen to increase the threshold that triggers a reaction in allergic patients. The mechanisms whereby OIT induces food tolerance are not completely understood, but it is clear that the treatment is not curative. Upon cessation of therapy, food allergy recurs within one to two months and continued ingestion of the food allergen is required to maintain the food unresponsive state. Interestingly, a hallmark of OIT is the strong induction of allergen-specific IgA and IgG antibodies, suggesting a possible inhibitory role for these antibodies in IgE-mediated food allergy reactions (Table 1) (13–21). The transient nature of these antibody responses would fit well with the temporary food tolerance induced by OIT.

**Table 1:** Summary of oral immunotherapy studies.

Ref	Study design	Sample size and age	Allergen	Allergen dose and duration	Efficacy outcome	IgE response	IgA/IgG response
(13)	Randomized, double-blind, placebo-controlled	*n* = 12 6–17 years	Cow's milk	500 mg milk protein for 23 weeks	Median milk threshold dose increased from 40 mg at BL to 5,150 mg after completion of OIT.	No change in CM-specific IgE.	↑ in CM-specific IgG4 (767%) from BL (*p* = 0.002).
(14)	Open-label	*n* = 11 7–11 years	Cow's milk	No maintenance phase; rush oral desensitization to 1,000 mg milk powder for 9 weeks with omalizumab followed by escalation up to 2,000 mg for 7–11 weeks	9 of 11 patients passed OFC 8 weeks after discontinuation of omalizumab treatment and began taking >8 g milk per day.	↑ in CM-specific IgE at weeks 36 (*p* < 0.05) and 52 (*p* < 0.01) compared to BL.	↑ in CM-specific IgG4 (15-fold) from BL at week 24 (*p* < 0.05).
(15, 16)	Open-label	*n* = 29 1–16 years	Peanut	1,800 mg PN protein for 36 months	93% passed 3.9 g OFC following completion of OIT.	↓ in PN-specific IgE levels by 12–18 months as compared to BL (*p* < 0.0005).	↑ in PN-specific IgG4 at 3 months from BL (*p* < 0.0005). Expansion of IgG4 following OIT was polyclonal and included *de novo* specificities (reduction in IgE binding occurred at the same epitopes that IgG4 binding gradually increased).
(17)	Randomized, double-blind, placebo-controlled	*n* = 69 1–4 years	Peanut	2,000 mg PN protein for 134 weeks; followed by 26 weeks of allergen avoidance	86% passed 5 g OFC at week 134.	Not reported.	↑ in PN-specific IgG4 levels at weeks 30, 82, and 134 as compared to BL (*p* < 0.01). ↑ in PN-specific IgA levels compared to BL at week 30 (*p* < 0.01); remained elevated throughout the course of OIT.
(18)	Open-label	*n* = 8 3–13 years	Egg	300–3,600 mg powdered EW for 18–50 months	75% passed 10 g OFC 1 month after stopping OIT.	↓ in EW-specific IgE levels as compared to BL (*p* = 0.03).	↑ in EW-specific IgG4 levels following OIT (*p* = 0.03). EW-specific IgG4 levels dropped at 6 months and 12 months after OIT was discontinued.
(19)	Open-label	*n* = 26 5–12 years	Egg	>1 hard-boiled EW for 1 year	16 of 26 subjects continued to ingest 1 or more hard-boiled EW during the 1-year maintenance phase. 5 subjects reduced the maintenance dose due to frequent allergic reactions.	↓ in EW-specific IgE levels at 3 (*p* < 0.01), 6 (*p* < 0.001), and 12 months (*p* < 0.001) compared to BL.	↑ in serum levels of EW-specific IgG4 at 12 months compared to BL (*p* < 0.001). ↑ in EW-specific IgG1, IgG3, and IgA levels rapidly at the rush phase compared to BL (*p* < 0.05) but gradually decreased throughout the maintenance phase.
(20)	Randomized, double-blind, placebo-controlled	*n* = 40 5–11 years	Egg	2,000 mg powdered EW. 22 months; followed by 2 months of allergen avoidance and 12 months of *ad libitum* allergen consumption	75% passed 10 g OFC at 22 months. 28% showed SU after discontinuation of OIT for 4–6 weeks.	↑ in EW-specific IgE levels for the first 3 months in OIT vs. placebo (*p* = 0.0001). EW-specific IgE in OIT group subsequently declined through the end of the study.	↑ in EW-specific IgG4 levels in OIT group compared to placebo (*p* < 0.0001). IgG4 levels rose sharply at 3 months and continued to increase until 36 months. ↑ in EW-specific IgA (*p* = 0.004), IgA1 (*p* = 0.005) and IgA2 (*p* = 0.047) in OIT group compared to placebo.
(21)	Open-label	*n* = 11 3–8 years	Egg	10 low-egg-allergen cookies (each containing 79–110 mg of EW protein) for 4 months	7 of 11 subjects could safely eat 0.5 g EW following OIT without an allergic reaction.	EW- and OM-specific IgE levels did not differ before and after OIT.	↑ in ratios of OM-specific IgG4/OM-specific IgE and IgA2/OM-specific IgE in OIT responders (*p* = 0.047).

↑, increase; ↓, decrease; BL, baseline; CM, cow's milk; EW, egg white; OFC, oral food challenge; OIT, oral immunotherapy; OM, ovomucoid; PBMC, peripheral blood mononuclear cells; PN, peanut; SU, sustained unresponsiveness.

The role of allergen-specific IgG antibodies as suppressors of allergic immune responses is well described (22–24). In brief, allergen-specific IgG is known to inhibit MC activation through two distinct mechanisms: (1) steric blockade of antigenic epitopes, rendering them invisible to MC-bound IgE, or (2) disruption of IgE:FcεRI signaling via the inhibitory Fc receptor, FcγRIIb. While the protective role of IgA is less well understood, there is emerging evidence that IgA, like IgG, can exert an inhibitory role in food allergy. This review will summarize current understanding of IgA and IgG antibodies as modulators of food allergy pathogenesis, specifically as is related to MC activation and effector function.

## Mast cells in food allergy

In mice, MCs are divided into two categories based on anatomical location and the types of proteases contained within their granules: mucosal MCs (MMCs) and connective tissue MCs (CTMCs). Murine MMCs are located in the epithelia of the intestinal and respiratory mucosa and preferentially express mouse MC protease (MMCP)-1 and -2. CTMCs, on the other hand, are found primarily around venules and nerve endings in connective tissue and express MMCP-4, -5, -6, and carboxypeptidase A (25). MC tissue distribution in humans is less clearly demarcated, as many human tissues have a mixed population of MC types. However, human MCs expressing both tryptase and chymase (MC_TC_) and those expressing tryptase only (MC_T_) are thought to correspond to murine CTMCs and MMCs, respectively (26).

On their surface, MCs express the high affinity IgE-receptor FcεRI. FcεRI exists in two isoforms: the trimeric αγ_2_ form and the tetrameric αβγ_2_ form, of which MCs express the later. The αβγ_2_ isoform is comprised of a single-membrane-spanning α-chain that binds the Fc portion of IgE, a tetra-spanning β-chain that amplifies the signal generated by the γ-subunit, and two γ-chains that exist as a disulfide-linked homodimer. Both the β- and γ-subunits contain immunoreceptor tyrosine-based activation motifs (ITAMs) in their cytoplasmic domains. An ITAM is a highly conserved sequence containing a tyrosine separated from a leucine or isoleucine by any other two amino acids. The subunits of the FcεRI complex have no known intrinsic enzymatic activity; instead, signaling occurs through the action of receptor-associated protein tyrosine kinases (PTKs) (27). After multivalent antigen recognition by FcεRI-bound IgE, adjacent FcεRI aggregate into lipid rafts rich in cholesterol, sphingolipids, PTKs, and GPI-anchored proteins (9). Following receptor aggregation, tyrosine residues within the ITAMs of the β- and γ- subunits are phosphorylated by β-chain receptor-associated LYN. Phosphorylation of ITAMs generates docking sites for SYK PTK and recruits additional LYN PTK (28). The resulting activation of SYK, following phosphorylation by LYN, induces a signaling cascade that drives many of the phenotypes of activated MCs, including degranulation (29). MC degranulation results in the release of preformed mediators (including histamine, neutral proteases, and TNF-α), rapid synthesis of pro-inflammatory lipid mediators (such as prostaglandins and leukotrienes), and the induction of genes encoding growth factors, cytokines, and chemokines (6).

The generation of a calcium signal is also critical for MC activation. Receptor-mediated activation of phospholipase C and the associated production of inositol 1,4,5-triphosphate (IP_3_) induces the release of Ca^2+^ from endoplasmic reticulum stores (30). The depletion of intracellular Ca^2+^ activates the influx of extracellular Ca^2+^ across the plasma membrane to replenish ER stores and sustain increases in cytosolic Ca^2+^ concentration (31).

In addition to inducing immediate hypersensitivity reactions, MCs also act as endogenous adjuvants for emerging type 2 adaptive immune responses and as inducers of type 2 inflammation. IgE-activated MCs serve as an important source of cytokines and chemokines associated with tissue inflammation and regulation of the adaptive immune response (32). Specifically, activated MCs are potent producers of the immunomodulatory cytokine, IL-4, which is critical for MC proliferation and survival as well as the enhancement of FcεRI expression (33). In the context of food allergy, MC-derived IL-4 is important for the induction of Th2 responses *in vivo* and acts directly on B cells to drive germline transcription and IgE isotype switching (10, 34).

The critical role of IL-4 in driving Th2 and suppressing regulatory T cell (Treg) responses in food allergy has been demonstrated using mice harboring a disinhibited form of the IL-4 receptor (*Il4raF709*). In these mice, a mutation in the IL-4 receptor α-chain immunoreceptor tyrosine-based inhibitory motif (ITIM) leads to amplified signaling upon interaction with IL-4 or IL-13. When repeatedly gavage-fed peanut butter in the absence of an adjuvant, *Il4raF709* mice become sensitized to peanut (as measured by the presence of serum peanut-specific IgE). Subsequently, following sensitization, exposure to large doses of peanut butter results in systemic anaphylaxis. In addition to strong IgE and Th2 responses, these mutant mice also have an expansion of MCs in the small intestine, which is lacking in their wild-type (WT) counterparts (35). However, in MC-deficient mice (*Kit^W−sh^*), these phenotypes are lost, even with the IL-4 receptor mutation (*IL4raF709 Kit^W−sh^).* These findings indicate that IL-4, working in concert with MCs, is necessary for the induction of allergen-specific IgE and Th2 cells in food allergy.

Intact IgE:FcεRI signaling is also required for the Th2 adjuvant function of MCs. In peanut-fed, *Il4raF709* mice treated with a SYK inhibitor to disrupt FcεRI signaling, specific Th2 cell responses and peanut-specific IgE levels are significantly reduced. In the same mice, however, robust induction of peanut-specific FoxP3+ Tregs is observed (36). Taken together, these studies provide clear evidence for the role of MCs in driving Th2 cell and IgE responses while suppressing Treg cell induction in food allergy.

## IgG overview

### Structure and function of IgG

IgG is the predominant immunoglobulin isotype in human serum and accounts for around 10%–20% of total plasma protein. Found in blood, lymph fluid, cerebrospinal fluid, and peritoneal fluid, IgG antibodies can neutralize toxins, viruses, and bacteria, opsonize them for phagocytosis, and activate the complement system (37). In humans, there exist four subclasses of IgG: IgG1, IgG2, IgG3, and IgG4. While the different subclasses of IgG are more than 90% identical at the amino acid level, each has unique properties with respect to antigen binding, immune complex formation, complement activation, triggering of effector cells, half-life, and placental transport (37). IgG1 is the most abundant IgG subclass in human sera, followed by IgG2, IgG3, and IgG4, respectively (37). IgG1 and IgG3 are formed primarily against protein antigens, whereas IgG2 is formed against repetitive T cell-independent polysaccharide structures found on encapsulated bacteria (38). Unlike the other subclasses, IgG4 does not mediate common IgG effector functions, including antibody-dependent cell-mediated cytotoxicity or complement dependent-cytotoxicity (9). Instead, IgG4 responses are largely restricted to non-microbial antigens and, like IgE, its production is dependent on help by Th2 cells (39). In contrast to human IgG subclasses, the murine IgG subclasses include IgG1, IgG2a/2c, IgG2b, and IgG3. The allotypes of IgG subclasses may differ between mouse strains; for example, IgG2c is found in C57BL/6 mice, while BALB/c mice have IgG2a but not IgG2c (40). Notably, murine IgG subclasses are functionally different to human IgG subclasses (41).

### IgG receptors on mast cells

In addition to the expression of the IgE-receptor, FcεRI, MCs also express Fcγ receptors (FcγRs) which bind IgG antibodies. Human MCs express FcγRI, FcγRIIa, and FcγRIIb, whereas murine MCs express FcγRIII and FcγRIIb (42). FcγRIIb, the only inhibitory IgG receptor, counteracts signals initiated by activating Fc receptors. It is a low affinity receptor that binds immune-complexed IgG and contains an ITIM in its cytoplasmic domain (43). Unlike activating receptors, the binding of IgG alone does not lead to inhibitory signaling via FcγRIIb. FcγRIIb is expressed conjointly with activating receptors on the surface of immune cells, and the inhibitory function of FcγRIIb requires a licensing signal by an activating receptor (9). As such, FcγRIIb cross-linking by immune complexes results in ITIM phosphorylation and inhibition of the activating signaling cascade.

### IgG in food allergy

There is convincing evidence to suggest that food-specific IgG antibodies play a major role in protection against food-induced reactions. For one, IgG antibodies are thought to contribute to natural protection from allergic reactions to food in patients with food allergen-specific IgE. Several studies demonstrate that natural resolution of milk allergy in children correlates with increasing IgG levels, specifically IgG4 (22, 44). Also, the presence of IgG4 in patients harboring peanut-specific IgE is associated with oral tolerance to peanut (45).

In addition to natural resolution of food allergy, the induction of IgG antibodies is likely to underlie the protective effects of OIT. Upon completion of OIT, patients can often ingest substantial amounts of the allergen without reacting, a condition referred to as food unresponsiveness. For example, in a study of peanut allergic patients who completed a 24-week peanut OIT regime, 67.5% of participants were able to ingest and tolerate six times more peanut protein without dose-limiting symptoms than at the start of treatment (46). To maintain the state of unresponsiveness following successful OIT, however, ongoing ingestion of the allergenic food is required. Interestingly, in patients who have completed OIT, levels of food allergen-specific IgE antibodies remain high. The observation that OIT does not change the concentration of existing allergen-specific IgE implicates the induction of an inhibitory factor that can suppress IgE-mediated anaphylaxis (9, 47).

**Figure 1:**
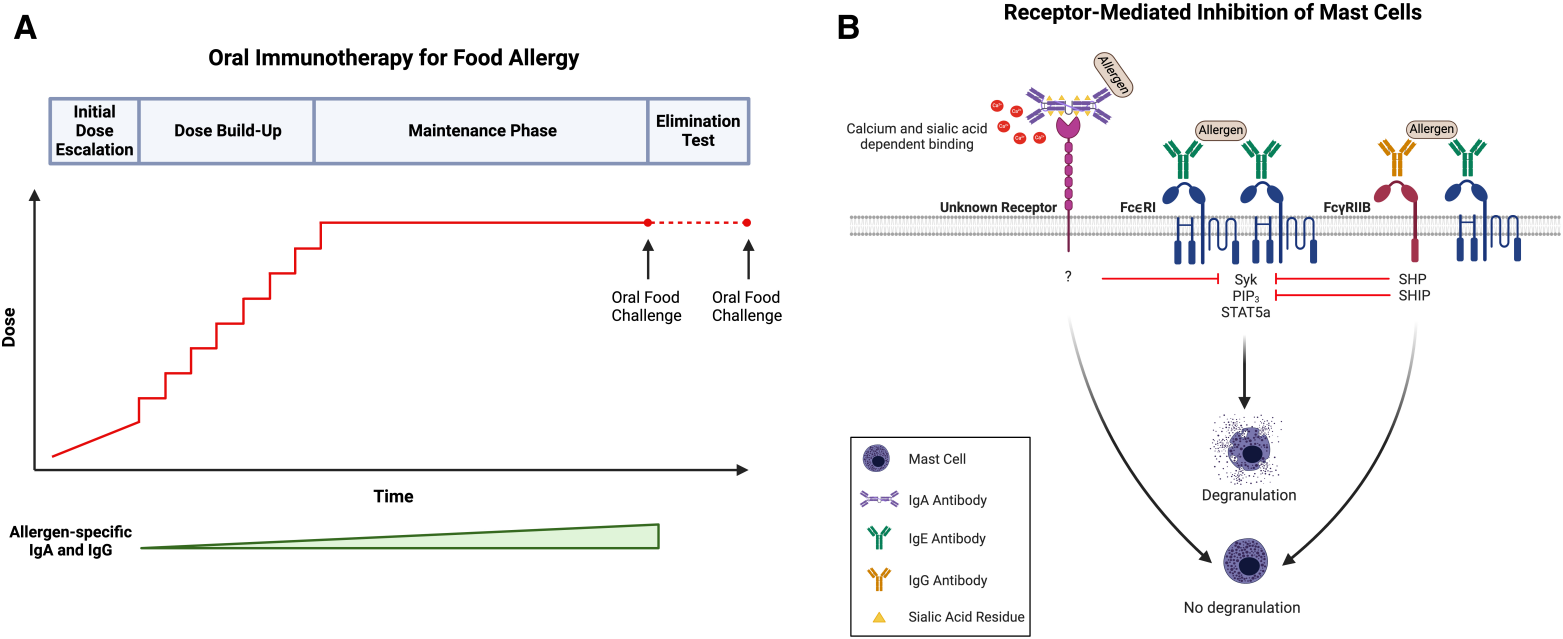
(**A**) Schematic of oral immunotherapy (OIT) protocol, including initial dose escalation, dose build-up, maintenance, and an elimination test to evaluate sustained unresponsiveness. Notably, both allergen-specific IgA and IgG levels increase over the course of treatment. (**B**) IgA- and IgG-mediated inhibition of mast cells (MCs). Antigen recognition by adjacent FcεRI-bound IgE on the surface of MCs (center) induces receptor crosslinking, phosphorylation of cytosolic immunoreceptor tyrosine-based activation motifs (ITAMs), and subsequent activation of several signaling pathways, including those involving SYK, PIP_3_, and STAT5a. Activation of these signaling intermediates drives many of the phenotypes of activated MCs, including degranulation. Following simultaneous allergen engagement by FcεRI-bound IgE and FcγRIIb-bound IgG (right), crosslinking of the two receptors leads to the phosphorylation of cytosolic immunoreceptor tyrosine-based inhibitory motifs (ITIMs). This results in the recruitment of protein tyrosine phosphatases (SHPs) and inositol phosphatases (SHIPs) which dephosphorylate the signaling intermediates induced by FcεRI activation, including SYK and PIP_3_, respectively. Downstream targets of STAT5a remain unaffected indicating that IgG:FcγRIIb downregulates transcripts involved in proinflammatory MC responses but not those involved in MC homeostasis. Additionally, antigen-specific IgA binds to MCs in a calcium- and sialic acid-dependent manner and blocks IgE-mediated MC activation (left). Like IgG, allergen-specific IgA inhibits the phosphorylation of SYK following IgE-receptor crosslinking; however, the exact receptor to which IgA binds as well as the mechanism by which IgA-binding leads to SYK dephosphorylation remains unknown. Created with BioRender.com.

There is a substantial body of evidence implicating allergen-specific IgG as the suppressive factor induced during OIT. Over the course of the treatment, levels of circulating food-specific IgG4 antibodies increase significantly in allergic subjects (Figure 1A) (13–21, 23). In a study by Jones et al*.,* peanut-specific IgE, IgG, and IgG4 was measured in the serum of 29 subjects over the course of OIT. They observed that peanut-specific IgG4 levels increased immediately following the start of treatment, reached statistical significance compared to baseline levels at three months, and remained elevated through the end of the study (15). Not only does OIT change the amplitude of the food antigen-specific antibody response, but also the specificity and diversity. While patients on allergen elimination diets have highly stable antibody repertoires, patients undergoing OIT experience dynamic and individualized changes in IgG specificity. In the sera of subjects undergoing OIT, there is an increase in the number of IgG epitopes and a simultaneous increase in IgG-binding intensity for many of the epitopes that existed prior to treatment (48). This finding indicates that the induction of IgG following OIT leads to both diversification of the allergen-specific IgG repertoire as well as increased concentration of individual IgG specificities. IgE epitope specificity, however, remains largely unchanged (16). Notably, IgG epitopes that emerge during OIT exhibit overlap with pre-existing IgE down to the amino acid level (16).

IgG antibodies formed in response to OIT are potent suppressors of IgE-mediated responses, including activation of MCs and basophils as well as systemic anaphylaxis (23, 45, 47, 49). The ability of IgG antibodies produced during OIT to suppress MC activation has been demonstrated *in vitro* using IgE-deficient (IgE^−/−^) *Il4raF709* mice (*Il4raF709* IgE^−/−^) that underwent enteral sensitization and subsequent OIT to ovalbumin (OVA) (23). As expected, sera obtained from the *Il4raF709* IgE^−/−^ mice failed to confer OVA sensitivity to WT bone marrow-derived MCs (BMMCs) as compared to sera from similarly sensitized, IgE-sufficient (IgE^+/+^) mice (23). BMMCs sensitized with a mixture of sera from both IgE^−/−^ and IgE^+/+^ mice showed a surprisingly weak response following OVA challenge, indicating a suppressive effect of the IgE^−/−^ sera (23). Depletion of IgG from the IgE^−/−^ sera abrogated its suppressive activity, implicating IgG antibodies as mediating the observed inhibition (23).

The inhibition of IgE-mediated hypersensitivity by IgG antibodies has been similarly demonstrated in food-allergic patients undergoing OIT. Using an indirect basophil activation test (iBAT), Burton et al. examined the capacity of serum from a cohort of peanut-allergic patients undergoing OIT to suppress basophil activation (23). Basophils from non-peanut-allergic donors were incubated with study sera and then exposed to peanut antigen. Expression of CD63, a granule protein rapidly induced following FcεRI crosslinking and closely linked to anaphylactic degranulation, was subsequently measured as a marker of basophil activation. Using this assay, they found that incubation with post-OIT sera led to decreased basophil activation by iBAT. They also observed less degranulation when donor basophils were incubated with a mixture of pre- and post-OIT serum as compared to incubation with pre-OIT sera alone. When donor basophils were exposed to IgG-depleted, post-OIT sera, however, this suppressive activity was diminished. Taken together, these findings suggest that food-specific IgG antibodies produced during OIT are capable of suppressing IgE-mediated hypersensitivity.

## IgA overview

### Structure of IgA

IgA is the most abundantly produced antibody isotype in the human body, mainly localized at mucosal surfaces. In fact, more than 80% of mammalian antibody secreting plasma cells (PCs) reside in the gut and express the IgA isotype (50). In humans, IgA exists in two isotypic forms, IgA1 and IgA2, with unique binding and signaling properties due to distinct glycosylation profiles (51). IgA1 is the predominant subtype, comprising 89% of serum IgA and 70% of jejunal fluid IgA (52). IgA1 differs from IgA2 due to the addition of 13 amino acids in its hinge region, a property which both facilitates antigen recognition and increases susceptibility to bacterial proteases (52).

Both monomeric and polymeric forms of IgA are found in the human body. While serum IgA is predominately monomeric, secretory IgA (SIgA), produced by plasma cells at the mucosal surface, is polymeric. More specifically, IgA in the intestinal mucosa exists as a dimer, linked by a cysteine-rich joining (J) chain, and becomes SIgA after secretion by the epithelial polymeric Ig receptor (pIgR) (52). Notably, a fragment of the pIgR, called secretory component (SC), remains covalently attached to SIgA, contributing to its stabilization, and allowing further modification by glycosylation. The highest levels of SIgA are generated and secreted at mucosal surfaces of the gastrointestinal, urogenital, and respiratory tract, while systemic IgA antibodies in the bloodstream are found at much lower concentrations (53).

### Function of IgA at the mucosal surface

Given their vast surface area and constant exposure to ingested and inhaled antigens, mucosal surfaces present major sites of vulnerability. IgA antibodies play a key role in maintaining intestinal microbial homeostasis. Through the process of immune exclusion, IgA antibodies prevent both dietary antigens and microbes from penetrating the epithelial surface (54). By removing potentially dangerous agents from the epithelial surface, IgA works to curb possible pathogenic immune responses. Studies have shown that pIgR-deficient mice lacking SIgA exhibit an increase in the uptake of both food antigens and commensal intestinal bacteria (55). Similarly, a report by Conrey et al*.* compared the translocation of live bacteria to the mesenteric adipose tissue (MAT) in IgA-deficient mice vs. IgA-sufficient littermate controls. Given that increased microbial translocation was observed in the IgA-deficient mice, their results suggest that IgA prevents translocation of commensal microbes to the MAT, thereby restraining the systemic anti-commensal IgG response (56). IgA also plays a role in regulating gut microbiota (57–61). Acting in a context-dependent manner, IgA can both prevent and promote bacterial colonization to control the composition of the microbiome. Low-affinity IgA contributes to the maintenance of commensal bacteria while high-affinity IgA binds to pathogens to promote clearance (62, 63).

### IgA in allergic disease

Several studies suggest a link between IgA and the development of allergic disease. In the clinical setting, much of the research exploring the relationship between IgA and atopy has examined patients with selective IgA deficiency (SIgAD), the most common form of primary immunodeficiency (64). Often times, SIgAD remains undiagnosed, given that more than 50% of patients are asymptomatic. However, symptomatic patients, particularly those with a complete absence of IgA, experience frequent viral and bacterial infections of the upper respiratory and gastrointestinal tracts as well as autoimmune and allergic disease (65). The true prevalence of allergy among SIgAD patients remains unclear, as studies from different countries present a variety of results. In a study conducted in Turkey, 45.7% of IgA-deficient patients presented with eczema, asthma, rhinitis, or atopic dermatitis; however, in a cohort of SIgAD patients in China, only 17% exhibited allergic symptoms (66, 67). This demonstrates the complexity and heterogeneity of IgA deficiency in terms of prevalence and clinical outcome.

A number of studies have demonstrated relationships between low IgA levels and the development of atopic disorders. Low levels of salivary IgA are associated with sensitization, allergic rhinitis, and atopic eczema, while high salivary IgA confers protection against allergy in sensitized infants (68–71). High fecal IgA is also associated with reduced risk of atopy (72). Taken together, these findings suggest a correlation between impaired IgA responses and the development of allergic disease.

### IgA and oral tolerance

Data examining the role of IgA and the development of food allergy in humans are limited; however, it has long been hypothesized that IgA mitigates allergic responses through immune exclusion of food antigens in the gut (73). As has been shown in the case of IgG, food-specific IgA antibodies increase dramatically after OIT (Figure 1A) (17, 19–21, 74). In a cohort of peanut-allergic children, those receiving peanut OIT experienced a marked increase in peanut-specific IgA in addition to peanut-specific IgG4, as compared to those receiving placebo OIT (17).

Elesela et al*.* recently demonstrated that IgA immune complexed to TNP/OVA can protect against Th2 responses in both the gut and lung. They also showed that IgA can bind to dendritic cells (DCs) to promote tolerance through the increased production of regulatory cytokines and the alteration of T cell activation. Murine bone-marrow derived DCs exposed to IgA immune complexes *in vitro* show an increased production of immunomodulatory cytokines, including IL-10 and TGFβ. Notably, these cytokines can both modulate the immune response directly as well as promote the development of Tregs (75).

Given that dysbiosis is likely a cause of food allergy, the role of IgA in promoting oral tolerance may also be attributed to its ability to regulate the microbiome (76). IgA antibodies in the gut have been demonstrated to moderate deleterious host responses to fluctuations in the microbiome and mucosal IgA deficiency leads to aberrant systemic exposures and immune responses to commensal bacteria (56, 77). Abdel-Gadir et al. found that both food-allergic infants and mice had decreased IgA and increased IgE binding to fecal bacteria. These findings suggest that dysbiosis in food allergy is associated with decreased SIgA responses and heightened Th2/IgE responses to the commensal flora (78). Food allergy susceptible *Il4raF709* mice had increased IgE^+^ bacteria in the gut following sensitization with OVA and Staphylococcal enterotoxin B, and challenge with OVA. Transfer of their microbiota to WT mice, increased susceptibility to food allergy in WT mice. When comparing food allergic children to healthy controls, Abdel-Gadir et al. identified bacteria from a variety of taxa associated with food allergy. Notably, the oral administration of human-origin *Clostridiales* species, related to the taxa impacted by dysbiosis in food-allergic infants, protected against food allergy in *Il4raF709* mice, normalizing the SIgA and suppressing the IgE responses to gut commensals (78). While these results clearly implicate the involvement of the gut microbiota in food allergy, the link between IgA, the microbiome, and oral tolerance is still not well understood.

Conflicting studies suggest that food-specific IgA does not correlate with natural tolerance to food antigens. Zhang et al*.* examined the allergen-specific IgA responses in mice following daily peanut exposure. After introducing peanut into the diets of WT C57BL/6 mice for 42 days, peanut-specific IgA was only detectable at low levels or not at all (79). Additionally, in a cohort of 441 atopic children, Liu et al*.* found the presence of fecal peanut-specific IgA in childhood is not a good predictor of protection from allergy later in life (80). While these findings appear to challenge the paradigm that the tolerogenic response to food antigens includes IgA production, it is important to consider that IgA responses vary significantly depending on several factors, including timing and location of sample collection as well as the atopic status of the patient (81).

## IgG as a suppressor of mast cell function in food allergy

Suppression of IgE-mediated MC activation by IgG occurs through at least two distinct mechanisms: steric blockade of immunodominant epitopes, and the much more sensitive mechanism of receptor-mediated inhibition via FcγRIIb (82). In steric blockade, IgG antibodies bind allergens in the extracellular space before they reach receptor bound IgE on the surface of MCs, rendering them invisible. By blocking IgE-binding epitopes, IgG antibodies effectively prevent the interaction between allergen and IgE, thereby inhibiting FcεRI-mediated MC activation. Notably, steric blockade requires high enough concentrations of IgG, along with a sufficient diversity of specificities, so as to cover all immunodominant epitopes. At high doses of IgG, inhibition of BMMC activation is observed even in MCs lacking the inhibitory IgG receptor confirming the contribution of a steric blocking effect independent of inhibitory IgG receptor signaling (23). Specifically, the IgG concentration required to mediate 50% inhibition of IgE-mediated activation is more than 10-fold higher for BMMCs lacking FcγRIIb (23). In receptor-mediated inhibition, the simultaneous engagement of an allergen by FcεRI-bound IgE and FcγRIIb-bound IgG leads to the phosphorylation of FcγRIIb cytosolic ITIMs. This triggers activation of phosphatases, such as Src homology 2 (SH2) domain-containing protein tyrosine phosphatase (SHP) and inositol phosphatase (SHIP), and the subsequent dephosphorylation of FcεRI signaling intermediates, including phosphoproteins (such as SYK) and phospholipids (such as PIP_3_) (9, 43) (Figure 1B). This mechanism does not require IgGs of broad specificity to cover a large number of epitopes on an allergen and is exerted at significantly lower concentrations of IgG.

### Physiologic effects of IgG on mast cells

It is well established that IgG can inhibit IgE-triggered MC degranulation in an FcγRIIb-dependent manner (23, 24, 83). Murine *in vitro* studies have shown that sensitized BMMCs from WT (FcγRIIb-sufficient) mice treated with antigen-specific IgG exhibit a marked reduction in LAMP-1 surface expression (a granule inner membrane protein, whose detection on the cell surface is used as a surrogate marker for MC degranulation) following IgE-mediated activation. However, in BMMCs derived from mice lacking FcγRIIb, this inhibitory effect is not observed at comparable doses of antigen-specific IgG (23, 83). In addition to inhibiting MC degranulation *in vitro*, IgG colligation can also inhibit IgE-mediated secretion of proinflammatory cytokines by BMMCs, including CCL2, IL-4, IL-6, IL-13, and TNFα (24, 83). Notably, this phenotype is lost in FcγRIIb-deficient BMMCs.

*In vivo* studies have shown that IgG antibodies can inhibit IgE-mediated anaphylaxis in mice through both epitope masking and FcγRIIb crosslinking (24, 83–85). Strait et al. suggested that, while both mechanisms contribute to the suppressive effects of IgG *in vivo*, inhibition of IgE-mediated anaphylaxis occurs predominantly through epitope masking at high concentrations of IgG and antigen (85). However, our own data and those of others suggest that suppression of anaphylaxis by IgG is primarily FcγRIIb-mediated. Multiple reports have shown that mice with a germline deletion of FcγRIIb experience more severe anaphylaxis in response to allergen challenge (24, 84). In a murine model of food allergy, the inhibitory effects of IgG administration are absent in mice lacking FcγRIIb (24). For example, in WT but not FcγRIIb-deficient mice, serum IL-4 levels and MC burden are decreased post challenge following IgG administration. Additionally, IgG administration in WT mice leads to increased Treg frequencies and suppression of Th2 polarization. However, in FcγRIIb-deficient mice Treg frequencies are reduced (24, 86, 87).

Our own investigations have nicely defined the role of MC-bound FcγRIIb in mediating the suppressive effects of IgG *in vivo.* In a murine model of passive systemic anaphylaxis, antigen-specific IgG treatment did not suppress antigen:IgE-induced hypothermia in mice harboring a MC-specific deletion of FcγRIIb as compared to FcγRIIb-sufficient mice (83). These findings confirm that the ability of antigen-specific IgG to suppress anaphylaxis is dependent on the expression of FcγRIIb specifically on MCs. We also examined the effects of IgG on IgE-induced MC transcriptional responses (83). Using RNA sequencing on BMMCs from WT and FcγRIIb-deficient mice, we identified genes activated following IgE-receptor crosslinking that were modulated in the presence of antigen-specific IgG acting via FcγRIIb. While many of the inhibited transcripts were identified as targets of SYK signaling, including immunoregulatory cytokines, chemokines, and growth factors, STAT5a-associated transcripts remained unaffected. Given the downstream pro-survival targets of STAT5a, including antiapoptotic factors MCL1 and BCL2, these findings suggest that IgG:FcγRIIb exerts a selective inhibitory effect on FcεRI-induced genes, downregulating transcripts involved in proinflammatory MC responses while leaving those involved in MC homeostasis unaffected.

## IgA as a suppressor of mast cell function in food allergy

Antigen-specific IgA antibodies have been shown to suppress IgE-mediated food allergy in mouse models of active and passive sensitization (88). Specifically, the systemic administration of antigen-specific IgA has been reported to suppress IgE-mediated anaphylaxis *in vivo*. In a model of passive sensitization, BALB/c mice pretreated with intravenous antigen-specific IgA experienced less severe hypothermia following oral challenge as opposed to those left untreated (88). The authors hypothesized that IgA treatment protected against anaphylaxis by inhibiting MC degranulation, given their MC dependent system. To test this hypothesis, serum levels of mouse MC protease 1 (MMCP-1), an enzyme released by degranulating MCs, were measured in mice injected with either antigen-specific IgA or saline. As expected, antigen-specific IgA treatment considerably reduced MC degranulation, and the detection of serum MMCP-1, in response to oral challenge (88).

While these observations clearly implicate IgA in the suppression of IgE-mediated MC activation, they do not clearly elucidate the mechanism by which IgA exerts this MC-specific effect. Some studies have concluded that IgA acts through steric blockage of the IgE epitopes on food allergen molecules, preventing recognition by MC bound IgE. Strait et al*.* showed that the suppressive effect of IgA is observed even in mice lacking the murine IgA receptor, Fcα/μR, and conclude that IgA confers protection by steric blockade rather than by a receptor-mediated inhibitory mechanism (88).

Our own investigations, however, suggest that IgA-mediated inhibition of MC activation might be receptor-mediated (Figure 1B). Given the protective role of IgA at mucosal surfaces, we hypothesized that IgA might be a potent regulator of MC function in the context of allergy. As is true for antigen-specific IgG, we found that sensitized BMMCs exhibit a marked suppression in LAMP-1 surface expression with the addition of antigen-specific IgA in culture (89). Our studies further established that IgA physically interacts with MCs by binding directly to both murine BMMCs and peritoneal MCs *in vitro.* Notably, IgA binding to BMMCs is dependent on calcium concentration and sialyation of the IgA antibodies. We demonstrated that the removal of exogenous calcium from BMMC suspensions prior to incubation with IgA dramatically impaired IgA binding (89). Additionally, treatment of IgA antibodies with neuraminidase to remove terminal sialic acid residues rendered IgA completely unable to bind to BMMCs. Desialylated antigen-specific IgA antibodies were also incapable of suppressing IgE-mediated BMMC degranulation as measured by LAMP-1 surface expression, suggesting that not only is sialylation required for binding but is also critical for the ability of IgA to suppress IgE-mediated MC activation (89).

Mechanistically, we demonstrated that antigen-specific IgA inhibits the phosphorylation of SYK following IgE-receptor crosslinking in BMMCs (89). Given that p-SYK is the most proximal signaling intermediate in the FcεRI signaling cascade, these results suggest that IgA exerts its inhibitory function at a receptor-proximal point and support a receptor-mediated inhibitory effect of IgA. While these findings provide evidence for a receptor-mediated effect of antigen-specific IgA on MCs, the relevant receptor mediating this effect remains unknown. Given that the association of IgA with MCs is dependent on both calcium and sialic acid, a member of the C-type lectin family of receptors may be mediating the inhibitory function of IgA. However, in mice with targeted deletions of SIGN-R1, CD33, and Siglec F, three members of the C-type lectin family known to be expressed on MCs, we have observed that IgA-mediated inhibition of IgE activated MCs remains intact (89). This is also the case in mice with a targeted deletion of FcγRIIb. Ultimately, additional receptor screening experiments will be required to fully elucidate the receptor-mediated mechanism by which IgA suppresses MC function.

IgA has also been shown to inhibit proinflammatory cytokine production following MC activation. The addition of antigen-specific IgA to sensitized BMMCs resulted in the complete suppression of pro-inflammatory cytokines, including IL-13, TNF-α, and IL-6, following stimulation (89). This finding indicates that IgA-mediated inhibition of MC effector function extends beyond degranulation to also affect cytokine production.

## Conclusion

Although not yet fully understood, the mechanisms underpinning the inhibitory role of IgA and IgG antibodies in food allergy represent potential targets for the development of novel therapeutics. As our understanding of these pathways continues to evolve, it seems promising that either the induction of IgA and/or IgG responses or the passive administration of allergen-specific IgA and IgG antibodies will be clinically beneficial to food allergic patients. Ultimately, further elucidating the role of these antibodies as regulators of MC effector function may provide insight into possible strategies for prevention and treatment of food allergy.
